# Treatment Outcomes for Elderly Patients over the Age of 70 with Early-Stage Peripheral Non-Small Cell Lung Cancer Who Were Treated with Stereotactic Body Radiation Therapy (SBRT) at a Total Dose of 55 Gy in Four Fractions: A Single-Institution Retrospective Study

**DOI:** 10.3390/jcm15145430

**Published:** 2026-07-10

**Authors:** Norio Mitsuhashi, Daichi Tominaga, Atsushi Motegi, Hajime Ikeda, Fumiya Shiina, Kazuhisa Kishimoto, Keiko Fukaya, Yoshitaka Nemoto

**Affiliations:** Department of Radiation Therapy, Hitachi, Ltd., Hitachinaka General Hospital, 20-1, Ishikawa-cho, Hitachinaka-shi 312-0057, Ibaraki-ken, Japan

**Keywords:** early-stage peripheral non-small cell lung cancer, stereotactic body radiation therapy (SBRT), very elderly patient, chest wall injuries

## Abstract

**Background/Objectives**: Due to its rapidly aging population, lung cancer is expected to become the second most common and deadliest cancer in Japan. Although surgery is the primary treatment for early-stage non-small cell lung cancer (NSCLC), advances in radiation therapy technology mean that stereotactic body radiation therapy (SBRT) is also a viable option for elderly patients with various underlying health conditions. **Methods**: We conducted a retrospective analysis to evaluate the outcomes of SBRT in 50 consecutive elderly patients (37 of whom were aged 80 and older) with early-stage peripheral NSCLC (Tis~T2aN0M0), who were treated with SBRT at our hospital and received a total dose of 55 Gy in four fractions. **Results**: The three-year overall, disease-free and cause-specific survival rates for all patients were 73.2%, 88.5% and 91.4%, respectively. For patients aged 80 years and older, these rates were 77.5%, 90.6% and 91.4%, respectively. There was no local recurrence. Hematogenous metastases were observed in four patients. However, hilar and subcarinal lymph node metastases developed in only one patient. Grade 2 pneumonitis and chest wall injuries (CWIs) were observed in two and five patients, respectively. Patients with larger tumors had a significantly higher incidence of chest wall injuries. **Conclusions:** SBRT at a total dose of 55 Gy in four fractions can achieve safe and satisfactory outcomes for early-stage peripheral NSCLC, even in patients aged 80 years and older. While CWIs were limited to Grade 2, attention to the chest wall dose is advisable when treating tumors adjacent to the chest wall.

## 1. Introduction

According to Japan’s 2025 cancer statistics projections, lung cancer is expected to be the second most common cancer and the deadliest cancer [1]. Due to advances in diagnostic technology and increased screening rates, the incidence of early-stage lung cancer has risen year after year. According to the Cancer Statistics IN JAPAN 2025, the incidence of stage I non-small cell lung cancer (NSCLC) increased to 42.2% in 2014~2015 [2]. It is suggested that the incidence has increased further recently.

The recommended treatment for early-stage NSCLC is surgical resection. However, the number of patients with comorbidities, such as respiratory failure or heart disease, as well as the number of elderly patients, is increasing. Conversely, significant progress has been made in radiation therapy planning and treatment equipment. These advances make it possible to apply stereotactic radiosurgery to tumors in the trunk, which was previously only used for intracranial lesions [3,4,5].

Therefore, stereotactic body radiation therapy (SBRT) is also used to treat early-stage NSCLC [6]. It has been demonstrated that the local control rate of SBRT is comparable to that of conventional radiation therapy, which involves a standard fractionation regimen of 60~70 Gy over 30~35 fractions. For stage I NSCLC, in particular, the incidence of solitary recurrence in the hilar region or mediastinum is low, so the value of prophylactic irradiation to these areas is limited. Consequently, SBRT, which concentrates high doses of radiation on the local tumor, has become one of the treatment options, and guidelines and nomograms have already been established [7,8,9,10]. Baker et al. propose a nomogram that can provide individual survival predictions for patients with early-stage NSCLC who are treated with SBRT in order to assist patients and clinicians in treatment decisions [11].

Many reports on SBRT for early-stage NSCLC have already been published. The study compared the efficacy of SBRT and surgical resection for peripheral NSCLC, and evaluated the optimal dose-fractionation regimen [7,8,12].

The Japan Clinical Oncology Group (JCOG) study JCOG0403 evaluated the safety and efficacy of SBRT in patients with stage IA NSCLC, using 48 Gy in four fractions. The 3-year overall survival was 59.9% and no severe toxicities were observed [13]. The author participated in this study. Based on the results of JCOG0403 and several phase II studies from Western countries showing that SBRT is effective and safe [13,14], the National Comprehensive Cancer Network and the Japan Lung Cancer Society recommend SBRT as a standard treatment option for patients with inoperable or refused stage I NSCLC [7,15]. Based on the results of JCOG0403, a randomized phase III trial (JCOG1408, or the J-SBRT trial) is underway to verify the superiority of increasing the total dose to 55 Gy in four fractions. The trial is comparing two dose-fractionation regimens of SBRT for medically inoperable stage IA NSCLC and small lung lesions that have been clinically diagnosed as primary lung cancer [16].

The population in Japan is aging rapidly. By fiscal year 2024, people aged 65 and older made up 29.3% of the population, and those aged 70 and older made up 23.4% [17]. Consequently, the number of elderly patients with a history of heart disease, vasculo-cerebellar disease, lung disease, or cancer is also growing. Furthermore, even among medically operable elderly patients, the number of those who do not wish to undergo surgery is growing.

Therefore, we analyzed the treatment outcomes in elderly patients over the age of 70—with a particular focus on those aged 80 years or older—with early-stage peripheral NSCLC who underwent SBRT with a total dose of 55 Gy in four fractions.

## 2. Materials and Methods

### 2.1. Eligibility Criteria

This study involved a retrospective review of the treatment outcomes for elderly patients over the age of 70 with early-stage peripheral NSCLC (Tis ~ T2aN0M0) who were treated with SBRT at a total dose of 55 Gy in four fractions. Patients were recruited from an institutional cohort database between January 2017 and December 2024. The final follow-up survey was conducted in March 2026. The SBRT eligibility criteria are: performance status (PS): 0~2; white blood cell count: ≥2000/μL; hemoglobin: ≥8.0 g/dL; platelet count: ≥3.0 × 10^4^/mm^3^; serum total bilirubin: ≤2.0 mg/dL; serum creatinine: ≤2.0 mg/dL; forced expiratory volume in one second (FEV_1_): ≥700 mL; blood oxygen saturation (SpO_2_): ≥90% (under room air); and sialylated carbohydrate antigen KL-6: ≤1000 μ/mL.

The Institutional Ethics Review Board approved this study (Study number BOE-38-001_202603-05). All participants provided written informed consent, and the study protocol was conducted in accordance with the World Medical Association’s Code of Ethics (Declaration of Helsinki).

### 2.2. Patients’ Characteristics

Table 1 shows the patients’ characteristics. Notably, 29 out of 50 patients (58.0%) were male. The mean age ± standard deviation (SD) of all patients was 82.5 ± 5.4 years (range: 71~95 years, median: 83 years). Those of male and female were 81.3 ± 5.3 years (range: 71~91 years, median: 82 years) and 84.1 ± 5.1 years (range: 72~95 years, median: 85 years). There were 37 very elderly patients aged 80 and older, 20 of whom were male and 17 of whom were female. Twenty-one patients (42%) had a history of other cancers, including seven patients (14%) with lung cancer.

The main reasons for the selection of SBRT instead of surgery are shown in Table 2. Only 15 patients (30%) chose SBRT, despite being medically operable. Thirteen patients (26%) were inoperable due to low respiratory function, such as COPD. Five patients (10%) were selected for SBRT due to a history of other cancers.

Although this was not the main reason for choosing SBRT, 21 patients (42%) had a history of cancer treatment, and seven of them (14%) had received treatment for lung cancer.

### 2.3. Tumors’ Characteristics

Table 3 shows the tumor characteristics. Regarding the T category, twenty patients had T1b disease, 13 had T1c disease, and 8 had T2a disease. Of the 50 patients, 20 (40%) had stage I A2 disease, and 13 (26%) had I A3 disease. The mean distance ± SD between the tumor and the chest wall in all patients was 8.1 ± 9.8 mm; in 19 out of these patients, the tumor was adjacent to the chest wall (range: 0~37.7 mm, median: 5.5 mm).

As shown in Table 3, twenty patients had tumors in the left lung. The upper lobe of the right lung was the most common location (17 out of 50 tumors), followed by the upper lobe of the left lung and the lower lobe of the right lung (11 out of 50 tumors).

A pathological and/or cytological diagnosis was confirmed in only 21 patients. The most common pathological type was adenocarcinoma (8 patients), followed by squamous cell carcinoma (7 patients). Among the patients with definitive pathological or cytological confirmation, 8 out of 13 cases (61.5%) were in patients under 80 years of age, and 13 out of 37 cases (31.5%) were in patients 80 years of age or older; the proportion was higher among patients under 80 years of age.

Twenty-nine patients were diagnosed solely based on clinical findings alone, including time-resolved chest computed tomography (CT) scans and/or fluorodeoxyglucose positron emission tomography (FDG-PET) without pathological confirmation. The final diagnosis of lung cancer in them was made after independent evaluation by respiratory physicians, thoracic surgeons, a radiologist, and two radiation oncologists, and was reached by unanimous agreement.

### 2.4. SBRT

#### 2.4.1. Patient Immobilization and Respiratory Restriction

The respiratory restriction technique (abdominal compression) was used to reduce tumor motion during respiration. During CT simulation, all patients were immobilized with a Vac-Lok^TM^ pad and HipFiX^R^. The cushion was placed at the level of the diaphragm to suppress respiratory motion. The pressure applied by the cushion was as high as the patient could tolerate.

#### 2.4.2. Clinical Target Volume (CTV) and Internal Target Volume (ITV) Delineation

For treatment planning, CT without contrast enhancement during three respiratory phases (normal shallow breathing, shallow hold inspiration, and shallow hold respiration) was performed to determine the ITV. CT was performed on 2 consecutive days to determine daily changes in the ITV. The gross tumor volume (GTV) was delineated in the normal shallow breathing phase on the first day using the lung window. No margins were added for the microscopic extension (CTV = GTV). The ITV was determined from the remaining 5 respiratory phases using CT images acquired over the course of two days. The ITV was generated by combining the 6 phase-sorted GTVs. To eliminate interobserver variability, the target volumes were delineated on the CT images by the same two radiation oncologists using Pinnacle version 9.10 (Koninklijke Philips N.V., Amsterdam, The Netherlands). All patients were treated with an ITV-based strategy with an additional ITV-to-PTV margin of 3 mm.

Previous reports have detailed the treatment plan and the assessment of respiratory movement; these should be referred to for further information [18].

#### 2.4.3. Treatment Planning

Treatment planning was performed using a three-dimensional radiation therapy planning system. A fixed multi-field irradiation technique with seven to eight non-coplanar static beams was used for 13 patients, while a multi-arc beam technique (Volumetric Modulated Arc Therapy: VMAT) with three to four arcs was used for the remaining 37 patients. The prescribed dose was 55 Gy in four fractions. This study prescribes to D95%, whereas the JCOG1408 protocols prescribe to the center [16]. Generally, 6MV high-energy X-rays were used for irradiation.

The planning organ at risk (OAR) includes the lung parenchyma, spinal cord, esophagus, pulmonary artery, stomach, intestines, trachea, bronchi, and other organs. The dose limits and permissible volumes were established in accordance with the JCOG0403 prospective clinical trial protocol [13] for “Phase II Trial of Stereotactic Body Radiation Therapy for T1N0M0 NSCLC”, in which the author conducted as a co-investigator at the JCOG while affiliated with a previous institution.

Prior to the start of treatment, two to three simulation irradiations were performed under intranasal oxygen administration at a flow rate of 2 L/min. This was done to verify treatment accuracy and to train the patient to take shallow breaths repeatedly during irradiation. After the treatment plan was finalized, the simulation irradiations were performed. The irradiation position was verified using cone-beam CT for each session.

Treatment was conducted over four consecutive days, beginning on either Monday or Tuesday and ending on either Thursday or Friday, to avoid spanning a weekend. Chemotherapy and/or immune checkpoint inhibitors were not administered to any patients.

### 2.5. Assessment of Tumor Control and Adverse Events

Assessment of local recurrence and/or distant metastasis was performed by a multidisciplinary team as described in Section 2.3.

Adverse events were evaluated using the Common Terminology Criteria for Adverse Events (CTCAE) version 5 system.

A comparative analysis was conducted on the dosimetric parameters, encompassing the minimum dose (V30), the maximum dose (Dmax), and the maximum dose to small volumes (D0.5 cm^3^, D1.0 cm^3^, and D5.0 cm^3^). This analysis was conducted between patients who exhibited chest wall injuries (CWI) and those whose tumors were in contact with the chest wall but did not manifest CWI.

### 2.6. Follow-Up

It was decided to follow-up with patients after treatment as follows: on their last day of treatment, after 2 weeks, and then 1, 3, 6, 12, and once every 6 months thereafter. Each visit included a comprehensive medical history and physical examination, blood tests (including biochemistry, complete blood count, and tumor markers), chest X-rays and/or CT scans, and a toxicity assessment. FDG-PET scans or abdominal CT scans were performed on some patients when local recurrence, lymph node metastasis, or distant metastasis was suspected.

The median follow-up period was 30 months, ranging from one to 107 months. A total of three patients with a follow-up period of four months or less were identified; they died from intercurrent diseases one month, three months, and four months, respectively, after SBRT.

### 2.7. Evaluation and Statistical Analyses

The survival curves were estimated using the Kaplan–Meier method. The log-rank test was then used to analyze the differences in survival curves. The incidences of chest wall injuries by T category and sex were compared using the chi-square test. A *t*-test was used to assess the distance between the tumor and the chest wall in patients with CWI compared to those without CWI. Statistical significance was set at *p* < 0.05. All statistical analyses and figures were generated using Microsoft^R^ Excel^R^ for Windows (version 2305) (Microsoft Corporation, Redmond, WA, USA), Statcel 4 (OMS Publishing Inc., Saitama, Japan), and R software (version 4.5.2; R Core Team).

## 3. Results

### 3.1. Survival Rates

Radiation therapy was administered according to the planned schedule in all patients, with no instances of interruption or extension to treatment.

**Figure 1:**
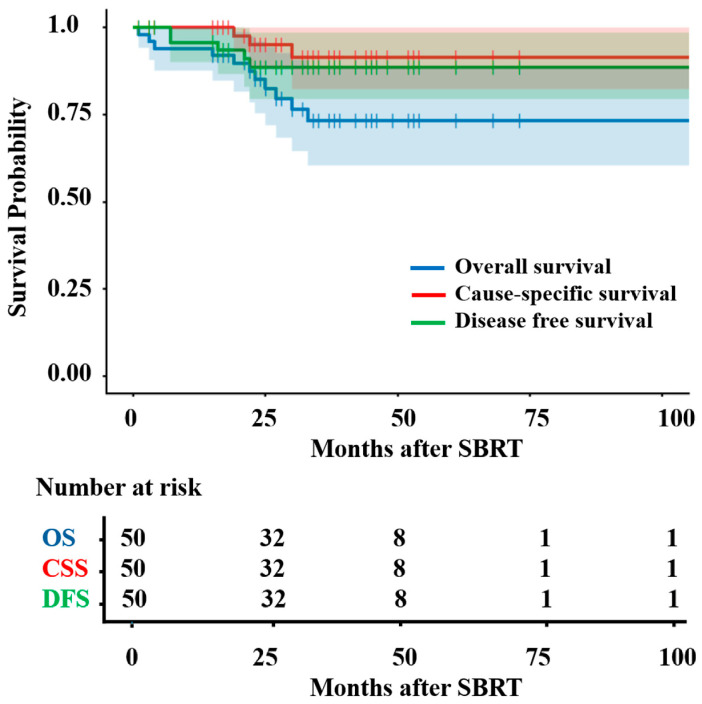
Overall, Disease-free, and Cause-specific survival curves for 50 patients with peripheral early non-small cell lung cancer treated with SBRT.

Figure 1 shows the overall, disease-free and cause-specific survival curves for all patients. The respective one-year survival rates were 94.0%, 95.7% and 100%. The respective 3-year survival rates were 73.2%, 88.5% and 91.4%. The 5-year survival rates were identical to the 3-year survival rates.

**Figure 2:**
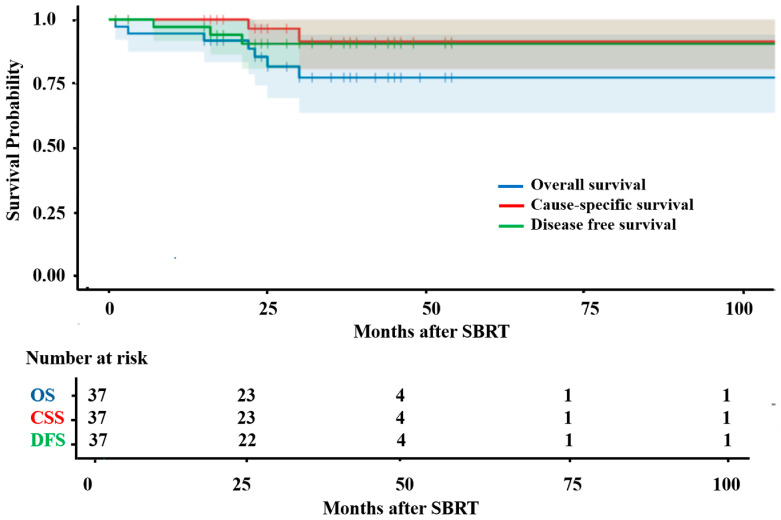
Overall, Disease-free, and Cause-specific survival curves for 37 Patients aged 80 and older with peripheral early non-small cell lung cancer treated with SBRT.

Figure 2 shows the overall, disease-free and cause-specific survival curves for 37 patients aged 80 and older. The respective one-year survival rates were 94.6%, 97.1% and 100%. The respective 3-year survival rates were 77.5%, 90.6% and 91.4%. The 5-year survival rates were the same as the 3-year survival rates.

**Figure 3:**
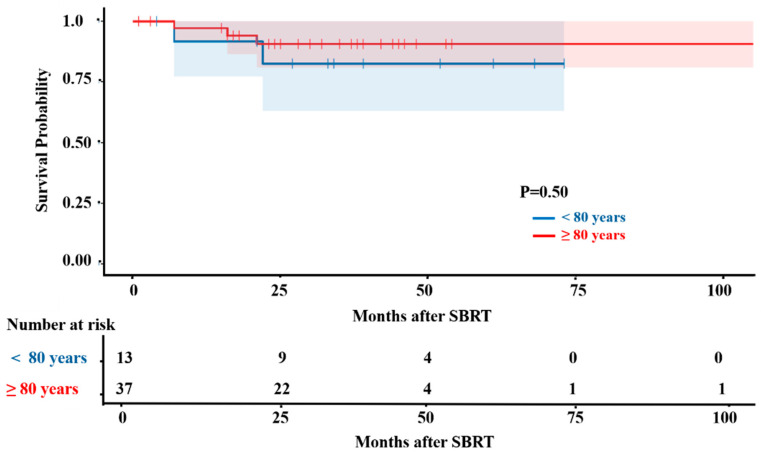
Disease-free survival curves according to patient age.

As shown in Figure 3, the three-year disease-free survival rates were 90.6% for patients aged 80 and older and 82.5% for those under 80. There was no significant difference between these rates (*p* = 0.50).

**Figure 4:**
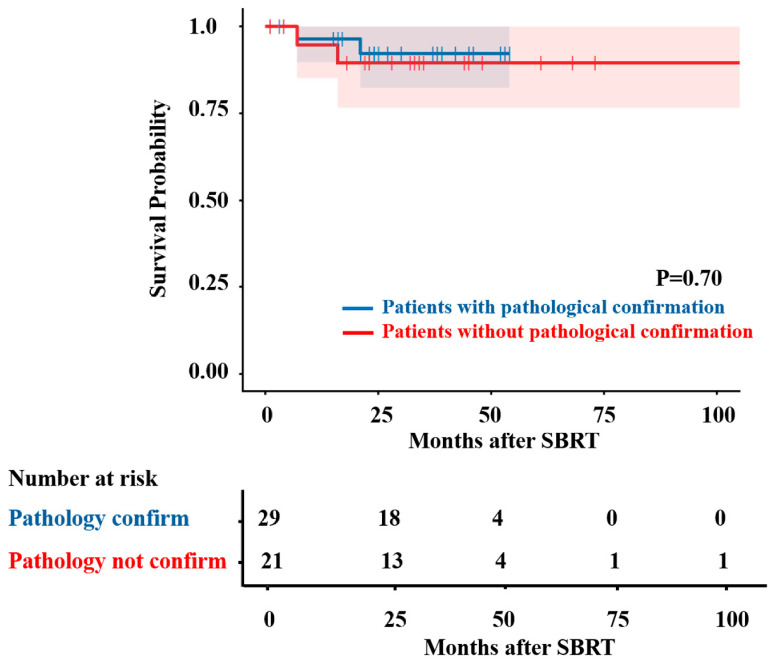
Disease-free survival curves according to pathological confirmation.

Figure 4 shows the disease-free survival curves according to pathological confirmation. The three-year disease-free survival rate was 92.3% for patients with pathologically confirmed tumors, compared to 89.4% for those with unconfirmed tumors (*p* = 0.70).

### 3.2. Recurrence Pattern

As shown in Table 4, there was no local recurrence, but hilar and subcarinal lymph node recurrence was observed in one patient seven months after SBRT. However, this patient had previously undergone surgery for squamous cell carcinoma of the right lower lobe (pStage IB) one and a half years earlier. Therefore, there is still a risk of lymph node recurrence from this cancer. Four patients developed distant metastases. One of these patients developed bone metastases after being treated with curative radiotherapy for adenocarcinoma of the right upper lobe (cStage IA3), involving a total dose of 60 Gy in 30 fractions. Therefore, there is also a possibility of bone metastasis from this cancer.

The treatment outcomes of SBRT for patients with early-stage NSCLC who are at least 80 years of age, as reported in international studies, are summarized and compared to current data in Table 5. According to reports from various institutions, the three-year cause-specific survival rate ranged from 70.8% to 93.7%, showing no difference from the findings of the current study. Furthermore, the three-year local control rate ranged from 71.4% to 90.1%.

### 3.3. Adverse Events

Adverse events were summarized in Table 6. Seven patients experienced Grade 2 adverse events: two patients with pneumonitis and five patients with CWI (one patient with rib fracture without subcutaneous induration or chronic dermatitis; two patients with rib fracture with subcutaneous induration; and two patients with chronic dermatitis with subcutaneous induration). All patients with CWI had tumors located close to the chest wall. Of the six patients with CWI, four were male (66.7%) and five were 80 years and older (83.3%). CWI was observed in 4 of the 37 patients (10.8%) who underwent SBRT using VMAT. Meanwhile, CWI occurred in 2 of the 13 patients (11.8%) who underwent SBRT using the fixed multi-field irradiation method; no difference in the incidence of CWI was observed between the two irradiation methods. There was no significant difference in the incidence of CWI between patients aged 80 and older and those under 80 (*p* = 0.578). The T category for the six patients who developed CWI was as follows: 3 out of 8 for T2a (37.5%), 1 out of 13 for T1c (7.7%), and 2 out of 20 for T1b (10%). The incidence of CWI was significantly higher in patients with a T2a tumor than in those with a combined T1b and T1c tumor (T1b + T1c vs. T2a: *p* = 0.041).

In the six patients in which CWI was observed, the mean and SD of the distance between the tumor and the chest wall were 0.37 mm ± 0.90 mm. A total of 14 patients were identified in whom the tumor was in contact with the chest wall despite the absence of CWI. The mean and SD of the distance between the chest wall and the tumor in the 44 cases without chest wall injuries, including these 14 patients, were 9.19 mm ± 10.0 mm. A statistically significant difference was observed between the two groups (*p* = 0.038). CWI occurred in 5 of the 19 cases (26.3%) in which the tumor was in contact with the chest wall.

Table 7 demonstrates the distance between the chest wall and the tumor, as well as dosimetric parameters such as the volume of the chest wall receiving ≥30 Gy (V30), the maximum dose (D max), and maximum dose to small volumes (D 0.5 cm^3^, D 1.0 cm^3^ and D 5.0 cm^3^), in six patients who developed CWI. With the exception of one patient in which the distance between the tumor and the chest wall was 2.2 mm, the tumors in the remaining five patients were in contact with the chest wall. The mean distance ± standard deviation (SD) between the tumor and the chest wall in six patients with CWI was 0.37 ± 0.82 mm. Conversely, the mean distance in 44 patients without CWI was 9.2 ± 10.0 mm, although in 14 out of these patients, the tumor was adjacent to the chest wall. The distance between the tumor and the chest wall in patients with CWI was found to be significantly shorter when compared with those without CWI (*p* = 0.038).

The mean V 30 Gy ± SD of the six patients was 45.26 ± 24.69 cm^3^ (range: 2.86~75.0 cm^3^). The mean Dmax ± SD of the patients was 63.00 ± 10.47 Gy (range: 40.3~71.9 Gy). D 0.5 cm^3^, D 1.0 cm^3^ and D 5.0 cm^3^ of the patients were 60.2 ± 11.4 Gy (range: 35.5~70.7 Gy), 59.0 ± 11.7 Gy (range: 33.8~69.9 Gy) and 52.9 ± 12.2 Gy (range: 27.5~64.9 Gy), respectively.

## 4. Discussion

In 2024, the average life expectancy for Japanese men is 81.09 years, and for women, it is 87.13 years. The average life expectancy for Japanese men aged 75 and 80 is 12.08 years and 8.96 years, respectively, while for women, it is 15.75 years and 11.83 years, respectively. Japanese people tend to live long lives. Men have a 25.8% chance of living to age 90, while women have a 50.2% chance [29]. For this reason, it is believed that early-stage lung cancer should be treated aggressively, even for the elderly, provided that safety can be ensured. Therefore, even the elderly patients aged 80 and older are candidates for curative treatment [6]. Until now, surgical resection has been the preferred treatment for early-stage peripheral NSCLC, even for elderly patients. However, since it has been established that SBRT yields comparable outcomes, there is a growing consensus that elderly patients with comorbidities should not undergo unnecessary surgery [30].

JCOG0702, titled “Phase I Trial of Stereotactic Body Radiation Therapy for T2N0M0 NSCLC in patients who are inoperable or elderly and refuse surgery”, confirmed that increasing the radiation dose to 13.75 Gy per fraction (for a total dose of 55 Gy) was safe, even for T2 cases. Consequently, our institution has adopted the total dose of 55 Gy in four fractions regimen since July 2016 [31]. Currently, a randomized controlled trial (JCOG1408: Randomized Controlled Trial of Stereotactic Body Radiation Therapy for Clinical Stage IA NSCLC or Isolated Lung Tumors of 3 cm or Less Diagnosed as Clinically Primary Lung Cancer (Unresectable Cases and Cases Refusing Surgery) is underway to verify that the total dose of 55 Gy in four fractions regimen (equivalent to JCOG0403) is superior to the standard treatment of the total dose of 48 Gy in four fractions (also equivalent to JCOG0403) [16].

To compare the effects of various treatment protocols with different fraction sizes and total doses, Biological Effective Dose (BED) was utilized in a linear-quadratic model. BED was defined here as nd (1 + d/α/β), with units of Gy, where n is fractionation number, d is daily dose, and α/β is assumed to be 10 for tumor tissue and for acute adverse effects of normal tissues. For chronic adverse effects of normal tissues, α/β is assumed to be 3 [32]. Ohnishi et al. reported preliminary results for a Japanese multi-institutional review of 257 patients with stage I NSCLC treated with SBRT. The results showed that local control and survival rates were better with BED ≥ 100 Gy than with <100 Gy, and survival rates were much better for medically operable patients than for medically inoperable patients [33]. The BED in total dose of 55 Gy in four fractions, which was employed in the current report, is 55(1 + 13.75/10) = 55(1 + 1.375) = 55 × 2.375 = 130.625.

The current study demonstrated that there was no difference in disease-free survival rate between patients aged 80 and older and those under 80 years of age. Some researchers have reported that SBRT is a safe and effective treatment for peripheral-type NSCLC in elderly patients. It has even been reported to be suitable for those aged 80 and older, and age is not an independent predictor of SBRT outcomes [24,25,28,34]. Bei et al. conducted a multivariate analysis of the outcomes of treatment for 153 patients aged 80 and older with stage I NSCLC who were treated with a total of four to six sessions of 10 to 13.75 Gy of SBRT and concluded that tumor size, pretreatment C-reactive protein levels, histologic type, and pretreatment PS were significantly associated with overall survival [34]. Palma et al. reported that the population-based study indicates that introduction of SBRT improves access to curative treatment for elderly patients, significantly reducing the number of patients who receive no treatment. They concluded that coincident with the implementation of SBRT, a significant improvement in survival was observed, which was limited to the patients treated with radiation therapy and not seen in patients receiving surgery or no treatment [6].

In the literature, the local recurrence rate for patients treated with SBRT has been reported to be less than 10% [35]. Although no patients in the current study who received a total dose of 55 Gy in four fractions developed local recurrence, caution should be exercised when comparing local control rates between reports. The local control rate can vary depending on where the prescribed dose evaluation points are set within the PTV, even when the total dose and number of fractions are the same [13,31].

A review of the literature comparing surgical outcomes with those of SBRT in patients aged 80 and older reached the following conclusion: Overall survival (OS) is generally slightly better with surgery due to differences in baseline performance status, but the treatment-related complication rate is clearly lower with SBRT, although there is no significant difference in cause-specific survival and local control rate. There is evidence suggesting that SBRT may offer advantages in terms of quality of life (QOL) and functional preservation [34,36,37,38].

Postoperative findings in patients with clinical stage IA NSCLC show that 15~20% of them have lymph node metastases [39,40,41,42]. Zhang et al. reported that lymph node metastases in NSCLC were strongly associated with age, smoking status, tumor size, histology and differentiation, carcinoembryonic antigen level, vascular invasion, and pleural invasion. The pattern of regional T1 peripheral NSCLC is significantly influenced by tumor size. Compared to primary tumor size, a large solid portion is a more clinical predictor of lymph node metastases in patients with clinical T1 partially solid lung adenocarcinoma [43].

In the current study, an 85-year-old female patient with a stage IA2 (T1b) tumor in the right lung in S1, which was not confirmed pathologically, was the only one to develop lymph node metastases. However, she had undergone surgical resection for squamous cell carcinoma of the right lower lobe (pStage IB) one and a half years prior to SBRT. The surgeon who operated on her and performed ongoing follow-up considered that the lymph node metastases originated from the carcinoma that was removed, but not the tumor that was treated with SBRT. Even when the lymph node metastases originated from a tumor that had been treated with SBRT, the incidence of lymph node metastases in the current study was low at 2% (one out of 50 patients), compared to reports from other postoperative studies [40,41,42,43]. This result could be interpreted as suggesting that the incidence of lymph node metastasis may be lower than that observed in surgical cases.

The current analysis revealed no statistically significant differences in disease-free survival (DFS) between patients aged 80 and older and those under 80 (*p* = 0.50), nor between patients with and without pathologically confirmed tumors (*p* = 0.70). However, given the relatively small sample size (n = 50) and the limited number of outcome events, the possibility of a Type II error cannot be ruled out. Consequently, collaborative, multi-institutional studies and prospective clinical trials with larger sample sizes are necessary to achieve a comprehensive and informed understanding of the matter.

As far as CWIs are concerned, the current study revealed that a tumor near the chest wall, tumor size and advanced age are risk factors. In the current study, the tumors were found to be in direct contact with the pleura or in close proximity to it, in cases where patients exhibited CWI. It cannot be ruled out that using the restricted breathing technique (abdominal compression) resulted in the chest wall receiving a higher radiation dose. As reported in the previous paper, the use of respiratory restriction (abdominal compression) to minimize the effects of tumor motion during SBRT may not have suppressed respiratory tumor movement adequately, and it cannot be ruled out that this led to increased radiation exposure to the chest wall [18]. This is because our previous research has already demonstrated a significant trend towards correlation between respiratory function and patient age in early-stage peripheral lung cancer (*p* = 0.061) [18]. Ma et al. analyzed a total of 57 studies incorporating 55,985 cases reporting clinical data on CWI after SBRT, and reported that the overall chest wall pain and rib fracture rates by Bayesian hierarchical modeling were 11.0% and 6.3%, respectively. The following factors are associated with an increased risk of chest wall toxicity after SBRT: sex (female > male), tumor-to-chest-wall distance (<16–25 mm), long contact length, Dmax, maximum dose of 0.5–5 cm^3^, and radiation exposure to the chest wall or ribs receiving > 30 Gy [44]. Voruganti et al. also reported the results of a systematic review. They concluded that rates of chest wall pain and rib fracture after SBRT are low; however, tumor location, accurate toxicity reporting, and dose-fractionation schemes might alter those rates [45]. Murakami et al., however, reported that the V30 of the chest wall, including rib, is significantly decreased in the VMAT plan. Especially irradiated volumes of rib were remarkably decreased in the VMAT plan, and the rate of the V30 for rib was reduced [46]. Ding et al. also reported that the VMAT plan significantly decreased the V30 compared to the static plan for the rib and chest wall [47].

The main limitations of the current study are its retrospective nature, the small size of the patient population, the inclusion of patients without pathological confirmation, and the modest median follow-up period at a single institution. Notably, since the study population includes patients for whom a histological diagnosis has not been confirmed, it is impossible to rule out the possibility of including benign lesions or indolent primary tumors. This carries the risk of overestimating local control and survival rates or underestimating recurrence rates. To some degree, this is to be expected in a patient population that has already outlived its average life expectancy. Ultimately, collaborative, multi-institutional studies and prospective clinical trials specifically designed for patients aged ≥ 80 years will be needed to strengthen the evidence base for this age group.

## 5. Conclusions

SBRT at a total dose of 55 Gy in four fractions can achieve safe and satisfactory outcomes for early-stage peripheral NSCLC, even in patients aged 80 years and older with various comorbidities. While chest wall injuries were limited to Grade 2, attention to the chest wall dose is advisable when treating tumors adjacent to the chest wall.

## Figures and Tables

**Table 1:** Characteristics of patients treated with SBRT.

Category	Item
No. of Patients	50
Sex (No. of Pts.)	Male: 29 Female: 21
Age ± S.D. (Yrs.)	Total: 82.5 ± 5.4
Sex (Yrs.)	Male: 81.3 ± 5.3 Female: 84.1 ± 5.1
Range (Yrs.)	71 ≤ Age ≥ 91 72 ≤ Age ≥ 95
Median (Yrs.)	82 85
Past history of other cancer (No. of Pts.)	21 (7: Lung cancer)

**Table 2:** Main reasons for selecting SBRT.

Reasons for Selecting SBRT	No. of Pts. (%)
Reduced lung function, such as in COPD	13 (26)
History of cerebral diseases, such as stoke	6 (12)
Complications of cardiovascular disease	6 (12)
Recent past history of other malignant tumors	5 (10)
Medically inoperable due to concomitant diseases	5 (10)
Medically operable but no desire for surgery	15 (30)

**Table 3:** Characteristics of tumors treated with SBRT.

Category	Item	No of Patients	%
Clinical Stage	0 (Tis)	2	4
(T category)	IA1	7	14
	(T1mi)	3	6
	(T1a)	4	8
	IA2 (T1b)	20	40
	IA3 (T1c)	13	26
	IB (T2a)	8	16
Location	Left lung	20	40
(Lobe)	Upper lobe	11	22
	Lower lobe	9	18
	Right lung	30	60
	Upper lobe	17	34
	Middle lobe	2	4
	Lower lobe	11	22
Pathology	Adenocarcinoma	8	16
	Squamous cell carcinoma	7	14
	Adeno-squamous carcinoma	1	2
	Non-small cell carcinoma	5	10
	No pathological confirmation	29	58

**Table 4:** Characteristics of patients and tumors in 5 patients treated with SBRT who developed recurrence.

Patient Sex (Age)	Tumor Location Laterality Segment	T Category	Stage	Pathology	Recurrence Site	Time of Recurrence (Month)
Male (84)	Left S8	1b	IA2	None	Liver and Lung	21
Male (82)	Right S3	1c	IA3	NSCLC	Lung	16
Male (77)	Left S5	1b	IA2	None	Lung	22
Female (77)	Right S10	1b	IA2	Adeno	Bone *	7
Female (85)	Right S1	1b	IA2	None	Hilar & Subcarinal Lymph nodes **	7

NSCLC: Non-small cell lung cancer, Adeno: Adenocarcinoma, None: No pathological confirmation. * A history of radiotherapy for adenocarcinoma of the right upper lobe (cStage IA3) at the same time. ** A history of surgery for squamous cell carcinoma of the right lower lobe (pStage IB) one and a half years ago.

**Table 5:** Summary of survival rates and local control rates in patients aged 80 and older with early-stage non-small cell lung cancer treated with SBRT.

Study	No. of Pts.	OS 1-Year 3-Years	DFS 1-Year 3-Years	CSS 1-Year 3-Years	LCR 1-Year 3-Years
Cassidy et al. [19]	58	NR 56.4	NR NR	81.6 72.6	71.4 71.4
Takeda et al. [20]	109	NR 53.7	NR 65.9	99.1 70.8	NR 82.3
Bei et al. [21]	153	NR 65.3	NR 58.0	98.0 75.7	100 (2-years)
Watanabe et al. [22]	64	88.9 68.3	81.0 58.9	98.4 93.7	94.8 90.1
Aoki et al. [23]	420	NR 76.0	NR	97.0 87.5	NR 79.2
Vorbach et al. [24]	118	89.7 NR	80.9 NR	97.3 85.8	99.1 NR
Kreibrink et al. [25]	31	59.2 (2-years)	NR	83.5 NR	100 NR
van Zyp et al. [26]	38	65 44 (2-years)	NR	NR	100 (2-years)
Sandhu et al. [27]	24	74 (2-years)	77 (2-years)	NR	100 (2-years)
Katano et al. [28]	205	93.9 70.7	83.3 55.0	NR	NR
Current study	37	94.7 77.5	97.1 90.6	100 91.4	100 100

NR: Not Recorded, OS: Overall Survival, DFS: Disease-Free Survival, CSS: Cause-Specific Survival, LCR: Local Control Rate.

**Table 6:** Adverse events of SBRT—Grading according to CTCAE ver. 5 -.

Adverse Events	Grade (No. of Pts.)	%
Pneumonitis	Grade 1 48	96
	Grade 2 2	4
Rib fracture	Grade 1 1	2
	Grade 2 1	2
Rib fracture with chronic dermatitis	Grade 2 2	4
Chronic dermatitis with induration of soft tissue	Grade 2 2	4

**Table 7:** Dosimetric parameters in 6 patients who developed chest wall injuries and 14 patients who did not, as well as distance between the tumor and the chest wall.

Patients	DBTC (mm)	CW Volume (cm^3^) *	V 30 Gy (cm^3^)	V 30 Gy (%)	D Max (Gy)	D 0.5 cm^3^ (Gy)	D 1.0 cm^3^ (Gy)	D 5.0 cm^3^ (Gy)
Patient with Chest Wall Injuries								
1 (G1) *	0	543.3	2.86	0.53	40.3	35.5	33.8	27.5
2 (G2) **	0	602.0	33.8	5.54	66.6	62.4	60.4	50.1
3 (G2) **	0	779.7	62.9	8.06	71.9	70.7	69.9	64.9
4 (G2) **	0	616.1	32.5	5.26	66.9	64.1	63.4	57.2
5 (G2) **	0	636.1	75.0	11.75	68.9	66.1	64.6	57.4
6 (G2) *	2.2	709.4	64.5	9.08	63.4	62.4	62.0	60.5
Patients without Chest Wall Injuries								
1 **	0	659.7	33.2	5.02	66.8	60.1	59.0	52.4
2 **	0	525.0	37.0	7.04	613	59.9	59.5	55.8
3 **	0	550.9	14.5	2.63	62.4	53.9	50.5	39.2
4 *	0	567.1	26.7	4.70	65.9	59.6	57.4	46.8
5 **	0	548.0	15.6	2.84	67.6	59.6	55.7	41.0
6 **	0	717.1	22.3	3.11	64.3	58.6	56.1	44.8
7 *	0	602.4	28.6	4.73	65.7	64.7	64.0	57.3
8 **	0	740.9	43.0	5.79	64.5	62.6	61.9	55.8
9 **	0	578.6	53.9	9.30	62.1	61.0	60.9	60.1
10 **	0	668.6	40.0	5.98	59.1	58.3	57.8	53.5
11 **	0	675.6	116.1	17.17	60.4	59.7	59.3	57.4
12 **	0	823.3	76.1	9.22	67.8	65.2	64.4	60.0
13 **	0	471.2	43.1	9.14	65.8	64.6	64.1	59.2
14 **	0	406.9	20.3	4.99	61.0	59.8	58.8	49.5

G1: Grade 1 chest wall injuries, G2: Grade 2 chest wall injuries. *: SBRT using a multi-arc beam technique. **: SBRT using the fixed multi-field technique. DBTC: Distance between the tumor and the chest wall. CW Volume in current study: Definition of chest wall (CW) is a 2-dimensional expansion of the ipsilateral lung located 2 cm above and below the planning target volume that excluded the lung volume, the mediastinal soft tissue, and the anterior vertebral body.

## Data Availability

The raw data supporting the conclusions of this article will be made available by the authors on request.

## Abbreviations

The following abbreviations are used in this manuscript.

NSCLC	Non-small Cell Lung Cancer
SBRT	Stereotactic Body Radiation Therapy
CWI	Chest Wall Injuries
JCOG	Japan Clinical Oncology Group
COPD	Chronic Obstructive Pulmonary Disease
PS	Performance Status
SD	Standard Deviation
CT	Computed Tomography
FDG-PET	Fluoro-Deoxy-Glucose Positron Emission Tomography
CTV	Clinical Target Volume
ITV	Internal Target Volume
GTV	Gross Target Volume
PTV	Planning Target Volume
OAR	Organ at Risk
CTCAE	Common Terminology Criteria for Adverse Events
VMAT	Volumetric Modulated Arc Therapy
BED	Biological Effective Dose
